# Dairy products intake and cancer mortality risk: a meta-analysis of 11 population-based cohort studies

**DOI:** 10.1186/s12937-016-0210-9

**Published:** 2016-10-21

**Authors:** Wei Lu, Hanwen Chen, Yuequn Niu, Han Wu, Dajing Xia, Yihua Wu

**Affiliations:** 1 Department of Toxicology, School of Public Health, Zhejiang University, 866 Yuhangtang Road, Hangzhou, People’s Republic of China; 2 Department of Epidemiology and Health Statistics, School of Public Health, Zhejiang University, 866 Yuhangtang Road, Hangzhou, People’s Republic of China; 3 The Second Affiliated Hospital, Zhejiang University School of Medicine, 866 Yuhangtang Road, Hangzhou, People’s Republic of China

**Keywords:** Dairy products, Cancer, Mortality risk, Dose–response, Meta-analysis

## Abstract

**Background:**

Dairy products are major components of daily diet and the association between consumption of dairy products and public health issues has captured great attention. In this study, we conducted a meta-analysis to investigate the association between dairy products intake and cancer mortality risk.

**Methods:**

After a literature search in PubMed and EMBASE, 11 population-based cohort studies involving 778,929 individuals were considered eligible and included in the analyses. Data were extracted and the association between dairy products intake and cancer mortality risk was estimated by calculating pooled relative risks (RRs) and corresponding 95 % confidence intervals (CIs). Sensitivity analyses and subgroup analyses based on regions, genders and dairy types were performed as well. Potential dose–response relationship was further explored by adopting the generalized least squares (GLST) method.

**Results:**

Total dairy products intake was not associated with all cancer mortality risk, with the pooled RR of 0.99 (95 % CI 0.92–1.07, *p* = 0.893). Subgroup analyses showed that the pooled RRs were 0.97 (95 % CI 0.92–1.03, *p* = 0.314) for milk, 0.88 (95 % CI 0.71–1.10, *p* = 0.271) for yogurt, 1.23 (95 % CI 0.94–1.61, *p* = 0.127) for cheese and 1.13 (95 % CI 0.89–1.44, *p* = 0.317) for butter in male and female, however the pooled RR was 1.50 (95 % CI 1.03–2.17, *p* = 0.032) for whole milk in male, which was limited to prostate cancer. Further dose–response analyses were performed and we found that increase of whole milk (serving/day) induced elevated prostate cancer mortality risk significantly, with the RR of 1.43 (95 % CI 1.13–1.81, *p* = 0.003).

**Conclusions:**

Total dairy products intake have no significant impact on increased all cancer mortality risk, while low total dairy intake even reduced relative risk based on the non-linear model. However, whole milk intake in men contributed to elevated prostate cancer mortality risk significantly. Furthermore, a linear dose–response relationship existed between increase of whole milk intake and increase of prostate cancer mortality risk.

## Background

Dairy products are major components of daily diet and due to their abundant nutrient elements such as protein, fat, minerals and vitamins, they are listed as core parts of dietary recommendation worldwide [1]. The proportion of dairy consumption was steadily increasing in several countries such as Japan since the past few decades [2]. Due to the large amount of dairy products intake in our daily life and their complex composition, the emerging role of dairy products has draw researchers’ attention extensively in public health.

It was universally acknowledged that dairy products intake was closely related to certain health issues. On the one hand, nutrients from dairy products were beneficial for chronic diseases such as cancer. For instance, casein was proved to have potential antimutagenic [3] and anticarcinogenic properties [4], while whey protein hydrolysate was demonstrated to protect against chemical-induced mammary tumor in rats [5]. On the other hand, some studies drew opposite conclusions. Park et al. confirmed that the milk protein casein promoted the proliferation of prostate cancer cells through in vitro assay [6]. Kroenke et al. harbored the view that high-fat dairy intake was associated with poor prognosis after breast cancer diagnosis, however no significant effect was found with respect to low-fat dairy intake [7]. Yang et al. demonstrated that among men with non-metastatic prostate cancer, post diagnostic dairy products intake increased prostate cancer-specific mortality risk and all-cause mortality risk [8]. In the aspect of cancer incidence risk, Huncharek et al. stated that higher consumption of milk or dairy products reduced colon cancer incidence risk [9], while Faber et al. suggested that dairy products increased risk of ovarian cancer modestly [10].

A few studies have conducted meta-analyses to investigate the correlation between dairy products intake and cancer incidence risk in colorectal [11], prostate [12], pancreatic [13], gastric [14] and ovarian cancers [15], nevertheless the relationship between dairy products intake and cancer mortality risk was diverse and inconsistent across individual studies, which has not been discussed systematically yet. Therefore, we conducted the meta-analysis to comprehensively explore this issue.

## Materials and methods

### Literature search

This meta-analysis was designed, conducted and reported according to PRISMA statements [16]. Systematic literature search was conducted in PubMed and EMBASE database up to May 2016. The following searching strategy was adopted in PubMed: “Dairy Products” [Mesh] AND “Neoplasms” [Mesh] AND (“survival” OR “mortality” OR “death” OR “HR” OR “RR” OR “OR” OR “hazard ratio” OR “relative risk” OR “odds ratio”), and similar strategy was adopted in EMBASE: ‘dairy’ AND (‘neoplasms’ OR ‘neoplasia’ OR ‘cancer’ OR ‘tumor‘OR ‘tumour’) AND (‘survival’ OR ‘mortality’ OR ‘death’ OR ‘hr’ OR ‘rr’ OR ‘or’ OR ‘hazard ratio’ OR ‘relative risk’ OR ‘odds ratio’). Only publications with full texts in English were taken into consideration. To avoid potentially missing studies during the primary search, the references of pertinent articles and relevant reviews were also scanned manually. The retrieved literatures were examined in detail to exclude potential duplications or repetitive data.

### Study selection

Duplicated studies were first excluded, then titles and abstracts were carefully scanned. Next full texts of potentially qualified studies were reviewed. We included studies if they met all the following criteria: (1) the studies of interest were dairy products intake; (2) the studies were population-based cohort studies and reported cancer mortality data; (3) relative risk (RR), hazard ratio (HR) or odds ratio (OR) estimates with 95 % confidence interval (CI) adjusted for multivariable factors were available or could be calculated; (4) original articles with full texts in English. Studies were excluded according to the following criteria: (1) reviews, letters, unpublished data or comments; (2) those published in languages other than English; (3) not population-based cohort studies; (4) RR, HR or OR estimates with 95 % CI were not available or could not be calculated.

### Data extraction

The study quality assessment was performed according to the Newcastle-Ottawa Scale [17]. Two reviewers (Dr. Yihua Wu and Dr. Wei Lu) extracted data using a standardized data extraction table independently. Any discrepancy was resolved by a third reviewer. Information extracted from each eligible study included the following items: first author, country, original study design, number of participants, gender, age, follow-up duration, dairy product types, group cut-off value, cancer types, endpoints, adjusted factors and study quality assessment. RR, HR or OR estimates with 95 % CI with regard to different types of dairy products and doses were recorded respectively. The most completely adjusted estimate was extracted if several risk estimates were available.

### Data synthesis and statistical analyses

The random-effect model was applied to calculate pooled RRs, 95 % CI and *p* value for heterogeneity. RRs comparing the highest intake category with the lowest intake category were combined across studies to generate the summary associations. The extent of heterogeneity across studies was examined using the I^2^ test [18] and I^2^ > 50 % together with *p* < 0.05 indicated significant heterogeneity. In order to validate the stability of outcomes in the meta-analysis, sensitivity analyses were performed by including studies which only reported all cancer mortality. Sequential omission of each individual study was also performed, while subgroup analyses were carried out to investigate the impact of regions, dairy product types and genders on cancer mortality. Funnel plots were constructed to assess the publication bias, meanwhile the Begg’s rank correlation test and Egger’s regression test was adopted to test the asymmetry and *p* < 0.1 indicated statistically significant publication bias [19].

We then looked for potential dose–response relationship between dairy products intake and cancer mortality risk using the generalized least squares(GLST) method for trend estimation of summarized data [20]. The doses reported in each study were first converted to servings/day, respectively. Kelemen’s study was excluded from dose–response analysis because dairy intakes were reported in densities (servings/1000 kcal). Bonthuis’s study was also excluded because dairy intakes were reported in g/day. The average of the lower and upper limits in each category were calculated and recorded as the mid-point dose. For open-ended intervals, we estimated the mid-point dose equaled to 1.5 times the lower limits. A potential curvilinear relationship was assessed using restricted cubic splines with four knots at fixed percentiles (5, 35, 65 and 90 %) of the distribution [20]. For model verification, we used *χ* ^2^ test and a *p* value for a non-linear relationship was calculated by testing the null hypothesis that the coefficient of the second spline was equal to zero. Non-linear model was applied in the first place if model verification indicated significance (*p* < 0.05), otherwise linear model was adopted. The dose–response curves containing RRs with 95 % CI for each dairy product type were constructed, respectively. Heterogeneity was tested using I^2^ test and I^2^ > 50 % together with *p* < 0.05 indicated significant heterogeneity.

All analyses were conducted using Stata software (version 13.0; StatCorp, College Station, TX, USA), and the significance level was set to *p* < 0.05 unless specified.

## Results

### Literature search

**Fig. 1:**
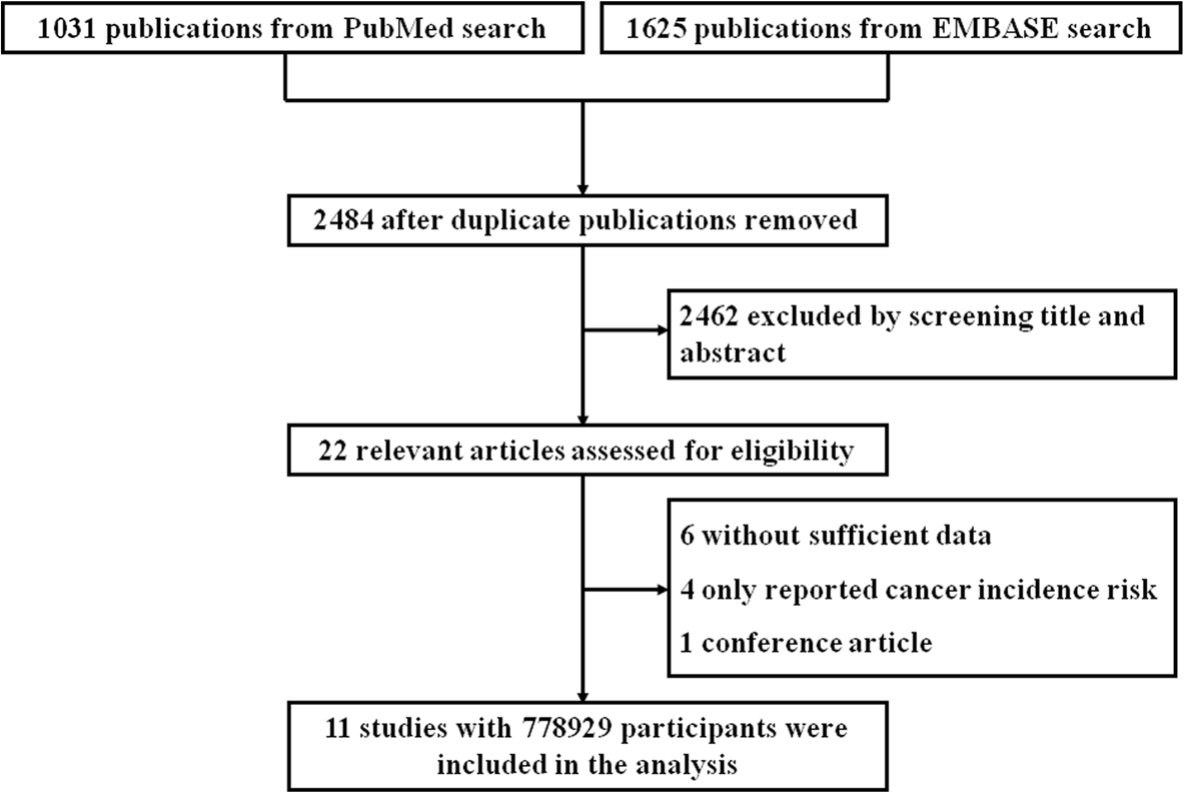
Flow diagram of the study selection process

We identified 1031 publications after searching PubMed and 1625 publications in EMBASE. First of all 172 duplicated studies were removed, followed by the exclusion of 2462 studies after reviewing abstracts and titles carefully. After full-text review of the remaining 22 articles, another 11 studies were excluded for the following reasons: six articles provided insufficient data, four only reported cancer incidence risk and one conference article. References of pertinent articles and relevant reviews were also scanned manually. Finally, the remaining 11 studies [21–31] with 778,929 participants were included in the following analyses (Fig. 1).

### Description of the included studies

Characteristics of the included studies were shown in Table 1. In brief, 11 studies were all population-based cohort studies, five were from America, three from Japan, one from Australia, one from Europe and one was multiethnic. However, Wang’s study and Kojima’s study were all from Japan Collaborative Cohort Study, and Wang focused on the correlation between milk consumption and all cancer mortality, while Kojima focused on the relationship between different types of dairy products consumption and colorectal cancer mortality. In addition, cancer types were various across studies. Dairy product types included total dairy, milk, yogurt, cheese, butter, whole milk and skim/low-fat milk. It was noteworthy that two studies reported prostate cancer, which was further discussed in the subgroup analyses. The quality assessment of each study was performed according to the Newcastle-Ottawa Scale, as shown in Table 2.

**Table 1:** Characteristics of the included studies

Study	Country of origin	Original design	Number of participants (Male/Female)	Age (years)	Follow-up (years)	Dairy products type	Group cut-off value
Bonthuis et al. (2010) [21]	Australia	Cohort	663/856	25–78	14.4	Total dairy	Mean: (163; 339; 628) g/day
Breslow et al. (2000) [22]	America	Cohort	8363/11641	18–87	8.5	Total dairy	(0–3.0; 3.0–7.0; 7.0–10.0; >10.0) servings/day
Chow et al. (1992) [23]	America	Cohort	17633/0	>35	20 (maximum)	Total dairy	(<46; 46–95; 96–142; >142) servings/month
Kelemen et al. (2005) [24]	America	Cohort	0/29017	55–69	15	Total dairy	Median: (1.0; 1.13; 1.24; 1.34; 1.45) servings/1000 kcal
Kojima et al. (2004) [25]	Japan	Cohort	45181/62643	40–79	9.9	Milk	(seldom; 0.5–4 servings/week; everyday)
						Yogurt	(seldom; 1–2 servings/month; 1–7 servings/week)
						Cheese	(seldom; 1–2 servings/month; 1–7 servings/week)
						Butter	(seldom; 1–2 servings/month; 1–7 servings/week)
Matsumoto et al. (2007) [26]	Japan	Cohort	4531/7075	19–93	9.2	Milk, butter and yogurt	(not everyday; everyday)
Park et al. (2007) [27]	America	Cohort	293888/0	50–71	6 (maximum)	Whole milk	(0; 0–0.5; 0.5–1; 1–2; > = 2) servings/day
						Low-fat milk	(0; 0–0.5; 0.5–1; 1–2; > = 2) servings/day
						Skim milk	(0; 0–0.5; 0.5-1; 1–2; > = 2) servings/day
						Cheese	(<0.1; 0.1–0.25; 0.25–0.5; 0.5–0.75; > = 0.75) servings/day
						Yogurt	(0; 0–0.5; > = 0.5) servings/day
Praagman et al. (2015) [28]	Europe	Cohort	8901/25508	20–70	15	Fermented dairy	Median: (8.8; 52.2; 128; 351) g/day
						Yogurt	Median: (3.8; 26.2; 62.9; 144.5) g/day
						Cheese	Median: (6.6; 19.6; 31.8; 53.2) g/day
Sharma et al. (2013) [29]	Multiethnic	Cohort	70333/76056	45–75	NA	Total dairy	(<=0.5; 0.6–1.0; 1.1–1.6; >1.6) servings/day
Song et al. (2013) [30]	America	Cohort	21660/0	40–84	28 (maximum)	Total dairy	(<=0.5; 0.5–1.0; 1.0–1.5; 1.5–2.5; >2.5) servings/day
						Whole milk	(<=1; 2–6; > = 7) servings/week
						Skim/low-fat milk	(<=1; 2–6; > = 7) servings/week
Wang et al. (2015) [31]	Japan	Cohort	39639/55341	40–79	19	Milk	(0; 1–2 servings/month; 1–2 servings/week; 3–4 servings/week; everyday)
Study	Cancer type	Endpoints	Adjusted factors				Quality assessment
Bonthuis et al. (2010) [21]	All cancer	All cancer death	Age, sex, total energy intake, body mass index, alcohol intake, school leaving age, physical activity level, pack years of smoking, dietary supplement use, b-carotene treatment during trial and presence of any medical condition				9
Breslow et al. (2000) [22]	Lung cancer	Lung cancer death	Age, sex, smoking duration and packs per day smoked				8
Chow et al. (1992) [23]	Lung cancer	Lung cancer death	Age, smoking status and industry/occupation				8
Kelemen et al. (2005) [24]	All cancer	All cancer death	Age, total energy, carbohydrate, saturated fat, polyunsaturated fat, monounsaturated fat, trans-fat total fiber, dietary cholesterol, dietary methionine, alcohol, smoking, activity level, body mass index, history of hypertension, postmenopausal hormone use, multivitamin use, vitamin E supplement use, education and family history of cancer				6
Kojima et al. (2004) [25]	Colon and rectal cancer	Colon and rectal cancer death	Age, family history of colorectal cancer, body mass index, frequency of alcohol intake, current smoking status, walking time per day, and educational level				9
Matsumoto et al. (2007) [26]	Colon, stomach, lung, liver, pancreatic, bile duct and blood cancer	Colon, stomach, lung, liver, pancreatic, bile duct and blood cancer death	Age and sex				9
Park et al. (2007) [27]	Prostate cancer	Prostate cancer death and advanced prostate cancer	Age, race, education, marital status, body mass index, vigorous physical activity, smoking, alcohol consumption, history of diabetes, family history of prostate cancer, screening for prostate cancer by use of prostate-specific antigen, intakes of tomatoes, red meat, fish, vitamin E, alpha-linolenic acid and total energy				8
Praagman et al. (2015) [28]	All cancer	All cancer death	Age, sex, total energy intake, smoking habit, body mass index, physical activity, education level, hypertension at baseline, intakes of alcohol and energy-adjusted intakes of fruit and vegetables				9
Sharma et al. (2013) [29]	All cancer	All cancer death	Time on study, years of education, energy intake, smoking behaviors, body mass index, physical activity, history of diabetes, alcohol intake, history of hormone replacement therapy, and history of oophorectomy				8
Song et al. (2013) [30]	Prostate cancer	Prostate cancer death	Age, cigarette smoking, vigorous exercise, alcohol intake, race, body mass index, baseline diabetes status, red meat consumption, total energy intake from recorded food items, assignment in the original aspirin trial and assignment in the original β-carotene trial. In addition, the models for whole milk and skim/low-fat milk were mutually adjusted for each other				8
Wang et al. (2015) [31]	All cancer	All cancer death	Age categories, smoking status, drinking status, physical activity, sleeping duration, body mass index, education level, participation in health checkups, green-leafy vegetable intake, and history of hypertension, diabetes and liver disease				9

**Table 2:** Quality assessment according to Newcastle-Ottawa Scale

Study	Q1	Q2	Q3	Q4	Q5	Q6	Q7	Q8	Total
Bonthuis et al. (2010) [21]	1	1	1	1	2	1	1	1	9
Breslow et al. (2000) [22]	1	1	1	1	2	1	1	0	8
Chow et al. (1992) [23]	1	1	1	1	2	0	1	1	8
Kelemen et al. (2005) [24]	0	1	1	1	2	0	1	0	6
Kojima et al. (2004) [25]	1	1	1	1	2	1	1	1	9
Matsumoto et al. (2007) [26]	1	1	1	1	2	1	1	1	9
Park et al. (2007) [27]	1	1	1	1	2	1	1	0	8
Praagman et al. (2015) [28]	1	1	1	1	2	1	1	1	9
Sharma et al. (2013) [29]	1	1	1	1	2	1	1	0	8
Song et al. (2013) [30]	0	1	1	1	2	1	1	1	8
Wang et al. (2015) [31]	1	1	1	1	2	1	1	1	9

### Association between total dairy products intake and cancer mortality risk

**Fig. 2:**
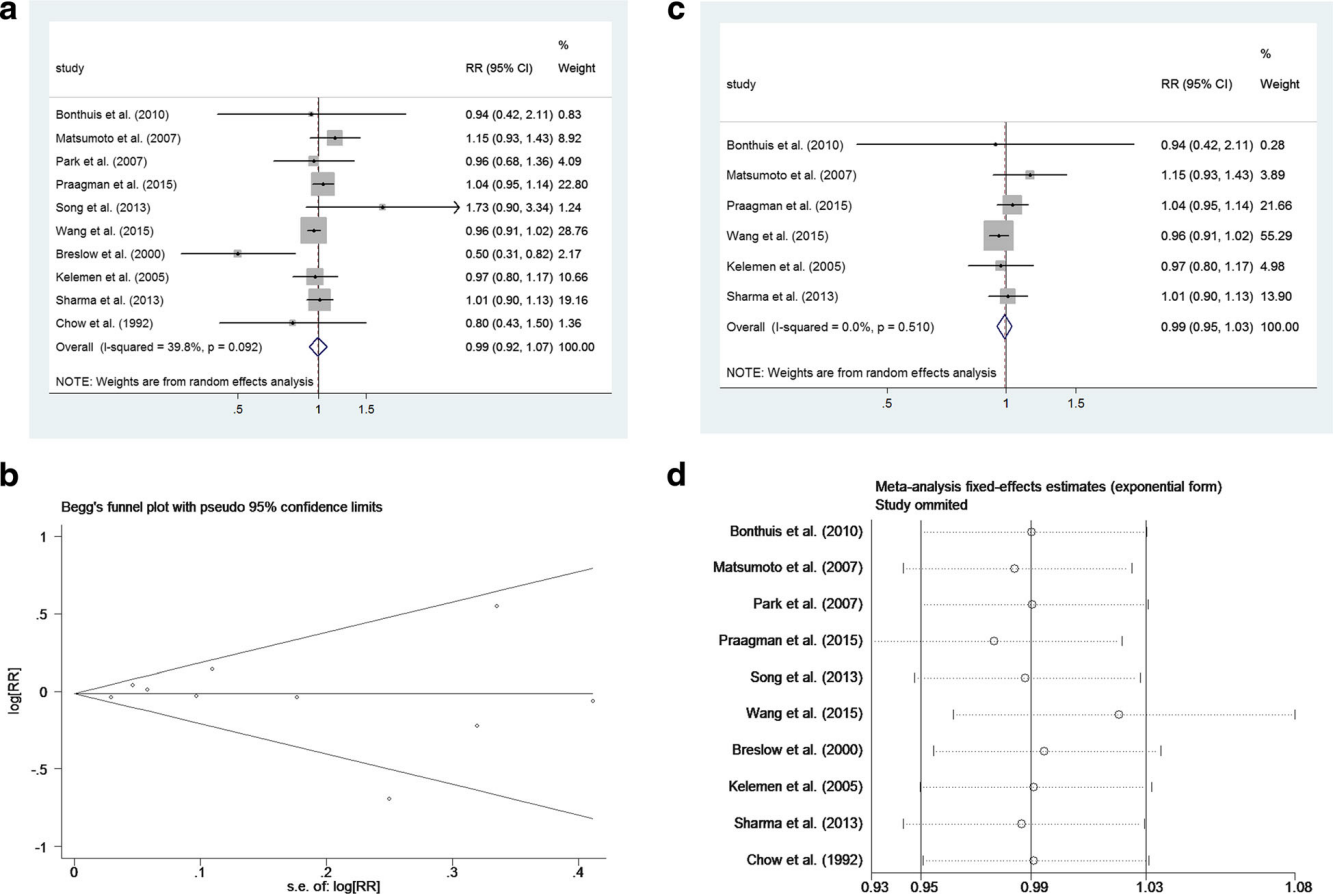
Total dairy intake and cancer mortality risk. **a** Forest plot of total studies evaluating relative risk of cancer mortality. **b** Begg’s funnel plot of total studies evaluating potential publication bias. **c** Sensitivity analysis was performed by including studies which only reported all cancer mortality. **d** Sequential omission of each individual study

In each individual study, RRs of the highest total dairy products intake group versus the control group were introduced. For the association between total dairy products intake and all cancer mortality, ten studies except Kojima’ study were included and the pooled RR was 0.99 (95 % CI 0.92–1.07, *p* = 0.893), as shown in Fig. 2a. No significant heterogeneity across studies was observed (I^2^ = 39.8 %, *p* = 0.092). Begg’s funnel plot and the Egger’s linear regression test were conducted to evaluate publication bias. The shape of Begg’s funnel plot showed no evident asymmetry (Fig. 2b), beyond that Egger’s test also suggested no publication bias existed (*p* = 0.947).

Sensitivity analyses were performed by including studies which only reported all cancer mortality (Fig. 2c), and the pooled RR was 0.99 (95 % CI 0.95–1.03, *p* = 0.679). Sequential omission of each individual study was also performed, as shown in Fig. 2d, the result pattern was not changed by removing single study each time.

### Subgroup analyses

Subgroup analyses were conducted according to different regions, dairy product types and genders. Initially, regions were categorized into America, countries other than America and Asia when we explored the association between total dairy intake and cancer mortality risk. We found the pooled RRs were 0.90 (95 % CI 0.67–1.21, *p* = 0.484) in America, 1.00 (95 % CI 0.95–1.04, *p* = 0.834) in countries other than America and 0.97 (95 % CI 0.92–1.02, *p* = 0.239) in Asia, which was in consistent with the above results.

Dairy product types were then categorized into milk, yogurt, cheese, butter, whole milk and skim/low-fat milk. In both genders, the pooled RRs were 0.97 (95 % CI 0.92–1.03, *p* = 0.314) for milk, 0.88 (95 % CI 0.71–1.10, *p* = 0.271) for yogurt, 1.23 (95 % CI 0.94–1.61, *p* = 0.127) for cheese and 1.13 (95 % CI 0.89–1.44, *p* = 0.317) for butter, proving that intake of these dairy products was not associated with cancer mortality risk significantly (Table 3). However, it was interesting to find that whole milk intake contributed to elevated cancer mortality risk significantly, with the pooled RR of 1.50 (95 % CI 1.03–2.17, *p* = 0.032), which was only limited to prostate cancer. In accordance with this finding, skim/low-fat milk intake was not associated with prostate mortality risk, with the pooled RR of 1.00 (95 % CI 0.75–1.33, *p* = 0.985).

**Table 3:** Subgroup analyses according to different dairy product types and genders

	Male and female				Male				Female			
	RR	95 % CI	Heterogeneity		RR	95 % CI	Heterogeneity		RR	95 % CI	Heterogeneity	
			I^2^ (%)	*p*			I^2^ (%)	*p*			I^2^ (%)	*p*
Total dairy	0.99	(0.92, 1.07)	39.8	0.092	1.00	(0.91, 1.11)	0.0	0.422	1.07	(0.96, 1.19)	0.0	0.393
Milk	0.97	(0.92, 1.03)	8.4	0.351	0.95	(0.89, 1.03)	35.1	0.214	NA	NA	NA	NA
Yogurt	0.88	(0.71, 1.10)	0.0	0.521	0.66	(0.42, 1.04)	0.0	0.757	NA	NA	NA	NA
Cheese	1.23	(0.94, 1.61)	0.0	0.985	1.19	(0.85, 1.67)	0.0	0.912	NA	NA	NA	NA
Butter	1.13	(0.89, 1.44)	1.0	0.315	NA	NA	NA	NA	NA	NA	NA	NA
Whole milk^a^	NA	NA	NA	NA	1.50	(1.03, 2.17)	0.0	0.963	NA	NA	NA	NA
Skim/low-fat milk^a^	NA	NA	NA	NA	1.00	(0.75, 1.33)	0.0	0.735	NA	NA	NA	NA

^a^cancer type was limited to prostate cancer

*NA* Not available

### Dose–response analyses

**Fig. 3:**
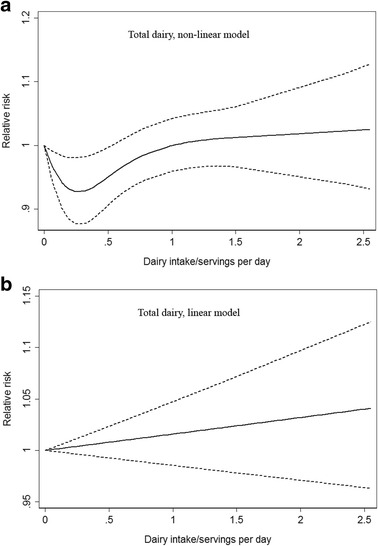
**a** Non-linear and (**b**) linear dose–response analyses for total dairy products intake and cancer mortality risk. Full lines represented RRs and dashed lines represented 95 % CIs

To begin with, the non-linear model between total dairy products intake and cancer mortality risk was constructed and *χ* ^2^ test was used for model significance verification, which revealed the existence of a non-linear association between them (*χ* ^2^ = 8.98, *p* = 0.030). The dose–response curves containing RRs with 95 % CI and doses were constructed (Fig. 3a), suggesting that low total dairy products intake may be protective against cancer related death, but high total dairy products intake did not have the same effect.

**Fig. 4:**
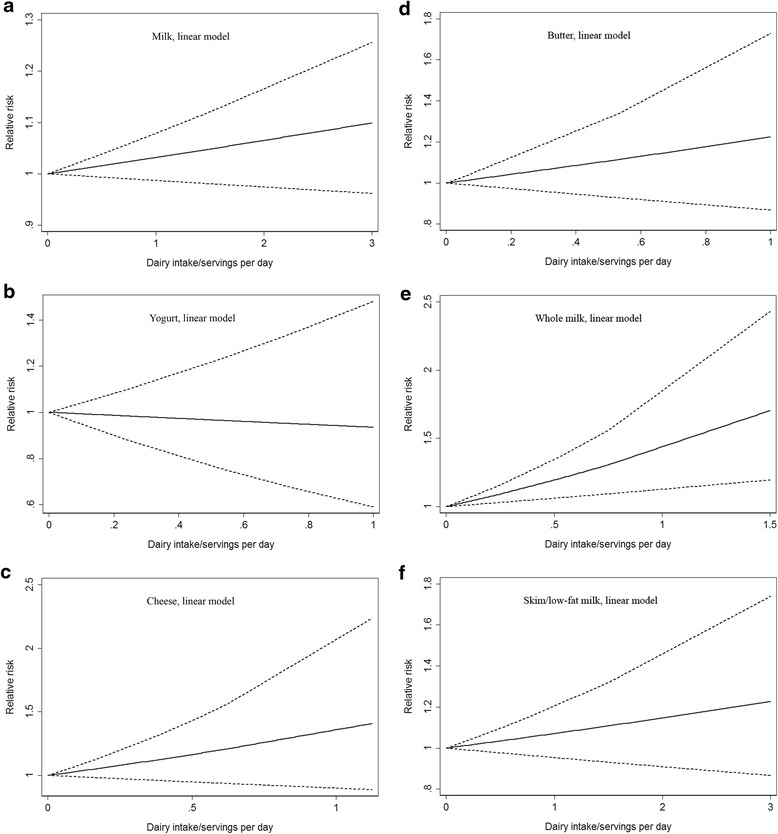
Linear dose–response analyses for (**a**) milk, (**b**) yogurt, (**c**) cheese, (**d**) butter, (**e**) whole milk and (**f**) skim/low-fat milk intake and cancer mortality risk. Full lines represented RRs and dashed lines represented 95 % CIs

For each dairy type, we adopted the linear model as well to assess RR due to increase of dairy products, which indicated that increase of total dairy, milk, yogurt, cheese, butter or skim/low-fat milk (serving/day) was not associated with elevated cancer mortality risk (Figs. 3b and 4 and Table 4). Nevertheless increase of whole milk (serving/day) contributed to elevated prostate cancer mortality risk significantly, with the RR of 1.43 (95 % CI 1.13–1.81, *p* = 0.003), which was in consistent with the previous subgroup analyses results.

**Table 4:** Dose–response analyses using the generalized least squares (GLST) method by adopting the linear model

	Male and female				Male				Female			
	RR per serving increase	95 % CI	Heterogeneity		RR per serving increase	95 % CI	Heterogeneity		RR per serving increase	95 % CI	Heterogeneity	
			I^2^ (%)	*p*			I^2^ (%)	*p*			I^2^ (%)	*p*
Total dairy	1.02	(0.99, 1.05)	33.8	0.334	1.00	(0.97, 1.04)	16.7	0.405	1.04	(0.99, 1.10)	7.7	0.564
Milk	1.03	(0.99, 1.08)	10.2	0.512	1.02	(0.97, 1.08)	8.7	0.275	1.05	(0.96, 1.14)	1.2	0.559
Yogurt	0.94	(0.59, 1.48)	5.1	0.409	0.60	(0.29, 1.26)	2.4	0.297	1.10	(0.51, 2.37)	0.1	0.715
Cheese	1.36	(0.90, 2.05)	5.3	0.260	1.23	(0.76, 1.99)	0.4	0.823	1.75	(0.79, 3.88)	4.3	0.037
Butter	1.22	(0.87, 1.73)	1.2	0.873	0.90	(0.45, 1.80)	0.1	0.738	1.27	(0.60, 2.71)	0.1	0.778
Whole milk^a^	NA	NA	NA	NA	1.43	(1.13, 1.81)	7.3	0.200	NA	NA	NA	NA
Skim/low-fat milk^a^	NA	NA	NA	NA	1.07	(0.95, 1.20)	0.3	0.877	NA	NA	NA	NA

^a^cancer type was limited to prostate cancer

*NA* Not available

## Discussion

Since dairy products contain complex nutrient composition and the amount of dairy products consumption is huge in our daily life, a number of studies have pointed out that dairy products may have impact on health issues such as obesity [32], diabetes [33, 34], cancers [10] and coronary heart disease [35, 36]. However, whether dairy products play a beneficial or detrimental role still remained controversial, largely depending on the types of dairy products and diseases. In view of this, we carried out this meta-analysis to comprehensively explore the association between dairy products intake and cancer mortality risk.

The current analyses showed that higher total dairy, milk, yogurt, butter and skim/low-fat milk intake was not associated with increased cancer mortality risk, while exposure to highest dose of whole milk intake increased about 50 % of prostate cancer mortality risk. By constructing a non-linear dose–response model, we concluded that low total dairy products intake may be protective against cancer related death, however high dose of total dairy products did not have the protective effect. Through a linear dose–response model, we found that increase of whole milk (serving/day) contributed to elevated prostate cancer mortality risk significantly, while other dairy types did not show the same effect. This might be explained by the hypothesis that luxuriant calcium contained in whole milk would increase the risk of prostate cancer by inhibiting the potential anti prostate carcinogenic nutrient 1,25-dihydroxyvitamin D [37]. Besides, high animal fat intake also contributed to poor prostate cancer mortality after diagnosis [38, 39]. However, although our meta-analysis shed new light on this issue, more future work remained to be done due to complex components of dairy products.

Our study had several crucial strengths. We conducted this thorough systematic search and applied comprehensive analytical approaches to assess the association between dairy products intake and cancer mortality risk. In addition, the studies we included were all population-based cohort studies of high quality. Furthermore, sensitivity analyses and sufficient subgroup analyses were also conducted to ensure the reliability of this study. Finally, we used a non-linear or linear model to fit the dose–response relationship between dairy products intake and cancer mortality risk. The methods of this study were rigorous and were based on guidelines for conducting the present study.

However, the current study was restricted by several limitations. First, the number of studies involved was relatively small, partly because cancer incidence risk rather than mortality risk was much more widely reported, thus the association between each type of dairy products and every specific cancer mortality risk was not available because of inadequate data. Second, most of the included studies were performed in Asia or America, and the studies conducted in America did not confine their cohorts to certain ethnic groups, hence the conclusions should be taken cautiously for other ethnic populations. We suggested further population-based cohort studies which investigate the association between dairy products intake and cancer mortality in each individual ethnic should be conducted. Finally a few studies reported different doses of highest dairy intake, which was further discussed in the dose–response analysis.

## Conclusions

On the basis of the results above, we confirmed that total dairy products intake was not associated with increased cancer mortality risk in both genders, yet low total dairy products intake even reduced relative risk based on the dose–response analyses. However, whole milk intake in men contributed to elevated prostate cancer mortality risk. Furthermore, the linear dose–response relationship existed between increase of whole milk intake and prostate cancer mortality risk.

## Abbreviations

CI: Confidence interval
GLST: Generalized least squares
HR: Hazard ratio
OR: Odds ratio
RR: Relative risk
